# Incorporation of Pharmacogenomics into Routine Clinical Practice: the Clinical Pharmacogenetics Implementation Consortium (CPIC) Guideline Development Process

**DOI:** 10.2174/1389200215666140130124910

**Published:** 2014-02

**Authors:** Kelly E. Caudle, Teri E. Klein, James M. Hoffman, Daniel J. Müller, Michelle Whirl-Carrillo, Li Gong, Ellen M. McDonagh, Katrin Sangkuhl, Caroline F. Thorn, Matthias Schwab, José A.G. Agúndez, Robert R. Freimuth, Vojtech Huser, Ming Ta Michael Lee, Otito F. Iwuchukwu, Kristine R. Crews, Stuart A. Scott, Mia Wadelius, Jesse J. Swen, Rachel F. Tyndale, C. Michael Stein, Dan Roden, Mary V. Relling, Marc S. Williams, Samuel G. Johnson

**Affiliations:** 1 Department of Pharmaceutical Sciences, St. Jude Children’s Research Hospital, Memphis, Tennessee, USA; 2 Department of Genetics, Stanford University, Palo Alto, California, USA; 3 Centre for Addiction and Mental Health, Toronto, Canada; 4 Department of Psychiatry, University of Toronto, Toronto, Canada; 5 Dr. Margarete Fischer-Bosch Institute of Clinical Pharmacology, Stuttgart, Germany; 6 Department of Clinical Pharmacology, Institute of Experimental and Clinical Pharmacology and Toxicology, University Hospital, Tuebingen, Germany; 7 Department of Pharmacology, University of Extremadura, Cáceres, Spain; 8 Department of Health Sciences Research, Mayo Clinic, Rochester, Minnesota, USA; 9 Laboratory for Informatics Development, NIH Clinical Center, Bethesda, Maryland, USA; 10 Laboratory for International Alliance on Genomic Research, RIKEN Center for Integrative Medical Sciences, Yokohama, Japan; 11 National Center for Genome Medicine, Institute of Biomedical Sciences, Academia Sinica, Taipei, Taiwan; 12 School of Chinese Medicine, China Medical University, Taichung, Taiwan; 13 Department of Medicine, Vanderbilt University, Nashville, Tennessee, USA; 14 Department of Genetics and Genomic Sciences, Icahn School of Medicine at Mount Sinai, New York, New York, USA; 15 Department of Medical Sciences, Clinical Pharmacology, Uppsala University, Uppsala, Sweden; 16 Department of Clinical Pharmacy and Toxicology, Leiden University Medical Center, Leiden The Netherlands; 17 Department of Pharmacology, Vanderbilt University, Nashville, Tennessee, USA; 18 Genomic Medicine Institute, Geisinger Health System, Danville, Pennsylvania, USA; 19 Department of Clinical Pharmacy, Skaggs School of Pharmacy and Pharmaceutical Sciences, University of Colorado, Denver, Colorado, USA; 20 Clinical Pharmacy Services, Kaiser Permanente Colorado, Denver, Colorado, USA

**Keywords:** Clinical practice guideline, guideline, pharmacogenetics, pharmacogenomics.

## Abstract

The Clinical Pharmacogenetics Implementation Consortium (CPIC) publishes genotype-based drug guidelines to help clinicians understand how available genetic test results could be used to optimize drug therapy. CPIC has focused initially on well-known examples of pharmacogenomic associations that have been implemented in selected clinical settings, publishing nine to date. Each CPIC guideline adheres to a standardized format and includes a standard system for grading levels of evidence linking genotypes to phenotypes and assigning a level of strength to each prescribing recommendation. CPIC guidelines contain the necessary information to help clinicians translate patient-specific diplotypes for each gene into clinical phenotypes or drug dosing groups. This paper reviews the development process of the CPIC guidelines and compares this process to the Institute of Medicine’s Standards for Developing Trustworthy Clinical Practice Guidelines.

## INTRODUCTION

The Clinical Pharmacogenetics Implementation Consortium (CPIC), a shared project between The Pharmacogenomics Knowledgebase (PharmGKB: www.pharmgkb.org) and the Pharmacogenomics Research Network (PGRN), was established in 2009 to address the need for practice guidelines that enable the translation of genetic laboratory test results into actionable prescribing decisions for specific drugs [1]. CPIC members are self-nominated and have expertise to contribute to the successful implementation of pharmacogenetic testing into patient care. CPIC membership now spans 12 countries and includes over 100 members from 58 institutions and multiple observers from the National Institutes of Health (NIH) and the Food and Drug Administration (FDA) (http://www.pharmgkb.org/page/cpic). At the time of this writing, CPIC has published nine gene-drug guidelines [2-10], two of which have recently been updated [11, 12]. In addition, four more updates and six new guidelines are in various stages of completion (http://www.pharmgkb.org/page/cpicGeneDrugPairs). An important CPIC activity has been the development of consensus guidelines that emphasize clinical relevance and applicability. The purpose of this manuscript is to review the CPIC guideline development process. We also compare this process to the Institute of Medicine’s Standards for Developing Trustworthy Clinical Practice Guidelines [13].

## CPIC GUIDELINE DEVELOPMENT PROCESS

CPIC guidelines are developed using established methods, including a rigorous review and grading of the relevant scientific literature, input of a writing committee composed of clinicians and basic researchers with expertise in the subject, a standard format (Table **1**) and an extensive pre- and post-submission peer review approval process. The CPIC Steering Committee and the CPIC Coordinator provide oversight for the guideline development process. 

### Underlying Assumption

The key underlying assumption for all CPIC guidelines is that clinical high-throughput and pre-emptive genotyping will eventually become common practice and clinicians will increasingly have patients' genotypes available before a prescription is written [1, 14-16]. Therefore, CPIC guidelines are designed to provide guidance to clinicians as to how available genetic test results should be interpreted to ultimately improve drug therapy, rather than to provide guidance as to whether a genetic test should or should not be ordered. The steps in the CPIC guideline development process are summarized briefly below.

### Initial Assessment of a Known Gene-drug Relationship

In 2009 and 2010 CPIC conducted two surveys, one among CPIC members and the other among members of the American Society for Clinical Pharmacology and Therapeutics (ASCPT). Responders were asked to rank 29 gene-drug pairs according to the perceived importance for using a genetic test to inform prescribing of the drug [1]. In 2012, this survey was repeated for CPIC members only. Gene-drug pairs selected for CPIC guidelines are prioritized based on several factors including the results of these surveys, the availability of strong supporting evidence for genetic variations that clearly affect a drug’s efficacy and/or risk of adverse reactions, clinically actionable prescribing recommendations, and the availability of genotype tests for use in the clinical setting. Moreover, strong consideration is given to whether reasonable alternative therapeutic strategies exist (*i.e. *changing dose or choice of therapy) with evidence in favor of their use, such that treatment could be improved for a patient whose genotype is known. The potential consequences of ignoring genetic test results versus using them to change therapy based on the probability of both the desired therapeutic effects and the undesired adverse effects are considered. Additional gene-drug pairs are considered after nomination by CPIC members or by external experts (who are then invited to join CPIC) and are identified by regular review of FDA recommendations and PharmGKB clinical annotations [17] of gene-drug pairs assigned a high level of evidence based on peer-reviewed literature. Nominations are discussed on regular, monthly CPIC conference calls and are approved by both CPIC members and the Steering Committee. Furthermore, the CPIC Steering Committee must approve the authorship plan, which includes a description of the writing committee, vetting for conflicts of interest, a writing plan with a timeline, and proposed prescribing recommendation(s) (see below for further details).

### Identification of Content Experts and Formation of Writing Committee

Once a guideline topic has been approved by CPIC members and the Steering Committee, a senior author is identified through self-nomination or by request of the CPIC Steering Committee. The senior author takes responsibility for forming the writing committee and completing the guideline. The writing committee is multidisciplinary, comprising a variety of scientists, pharmacologists, and clinicians (*e.g*., pharmacists and physicians). Authors will have a track record of publication and/or expertise in the specific topic area of the guideline. PharmGKB assigns at least one Scientific Curator to each CPIC guideline writing committee who has expertise in searching, compiling and evaluating the evidence for gene-drug associations, and making it computable and available on the PharmGKB website. Furthermore, PharmGKB curators often take primary responsibility for completing background gene and drug summaries, assigning likely phenotypes based on genotypes (*i.e. *“Table **1**” in guidelines), literature review, as well as preparing supplementary material provided in each guideline (*i.e.* genotypes that constitute the star (*) alleles or haplotypes, frequencies of alleles in major race/ethnic groups, genetic test interpretation and availability, and evidence linking genotype with phenotype).

The writing committee first determines whether it is likely that, based on available evidence, the therapeutic recommendation table (Table **2**) in the proposed guideline will contain specific recommendations for changing dose or choice of drug based on genotype. Without specific, actionable recommendations, the need for a CPIC guideline is reconsidered. If a guideline is not deemed appropriate, a new category of pharmacogenetic summary (an “evaluation”) can instead be considered (see sub-section “Development of Therapeutic Recommendation and Assignment of Strength of Recommendation” for further explanation).

### Management of Conflicts of Interest

All authors must declare any funding interests and activities potentially resulting in conflict of interest by written disclosure to the CPIC Steering Committee and writing committee before the approval of the authorship plan. Included are all possible conflicts including spouses/family members in declarations, NIH funding that could be interpreted to indicate that authors are “advocates” of the recommendations, as well as any sources of revenue from consulting, patents, stock ownership, etc. All conflicts of interest are reported in the guideline manuscript.

### Retrieval, Summarization and Presentation of the Evidence Linking Genotype to Drug Variability

The PharmGKB Scientific Curator, the CPIC coordinator or authors with experience in literature or systematic review conduct the literature review and present the results to the writing committee. A search of PubMed and OVID MEDLINE is performed using the keywords for the gene and drug of interest, for example: (gene name) OR (gene symbol) OR (dbSNP rs number) OR (gene common names) AND (drug name OR drug class name). Furthermore, papers listed on PharmGKB are cross-checked as there may be annotations for the papers and/or additional publications. Examples of types of evidence reviewed include, but are not limited to, randomized clinical trials with pharmacogenetic-based prescribing versus dosing not based on genetics, pre-clinical and clinical studies demonstrating that drug effects or concentration are linked to functional pharmacogenetic loci, case studies associating rare variants with drug effects, *in vivo* pharmacokinetic/pharmacodynamics studies for drug or reference drug plus variant type, and *in vitro* metabolic and/or transport capacity for the drug plus variant type. Where available, evidence evaluating the outcomes when prescribing has been altered based on genetic testing is included. For most gene-drug pairs, randomized controlled trials comparing clinical outcomes with genotype-guided dosing versus conventional dosing are not available. 

Publications supporting a major finding are usually considered as a group and scored by members of the writing committee based on the totality of the evidence supporting that major finding. Thus, it is possible for an evidentiary conclusion based on many papers, each of which may be relatively weak, to be graded as “moderate” or even “strong,” if there are multiple small case reports or studies that are all supportive with no contradictory studies. The rating scheme uses a scale modified slightly from Valdes *et al*. [18]: *high*, evidence includes consistent results from well-designed, well-conducted studies; *moderate*, evidence is sufficient to determine effects, but the strength of the evidence is limited by the number, quality, or consistency of the individual studies; generalizability to routine practice; or indirect nature of the evidence; *weak*, evidence is insufficient to assess the effects on health outcomes because of limited number or power of studies, important flaws in their design or conduct, gaps in the chain of evidence, or lack of information. Primary publications are summarized in the Evidence Table which is published in the manuscript supplemental material. It is the writing committee’s evaluation of this evidence that provides the basis for the therapeutic recommendation(s).

### Development of Therapeutic Recommendation and Assignment of Strength of the Recommendation

The writing committee discusses the evaluation of the literature and develops a draft recommendation via web conferences and email communication. CPIC’s therapeutic recommendations are based on weighing the evidence summarized in the supplementary Evidence Table from a combination of preclinical functional and clinical data, as well as on any existing consensus guidelines [1]. Evidence related to the suitability of alternative medications or dosing that may be used based on genetics must be weighed in assigning the strength of the recommendation. Overall, the therapeutic recommendations are simplified to allow rapid interpretation by clinicians and are presented in the Table **2** of each guideline and occasionally in an algorithm. 

To assign strength to a recommendation, CPIC uses a transparent three category system for rating recommendations that was adopted with slight modification from the rating scale for evidence-based recommendations on the use of antiretroviral agents (http://aidsinfo.nih.gov/contentfiles/AdultandAdolescentGL.pdf). Therapeutic recommendations are graded as: *strong*, where “the evidence is high quality and the desirable effects clearly outweigh the undesirable effects”; *moderate*, in which “there is a close or uncertain balance” as to whether the evidence is high quality and the desirable clearly outweigh the undesirable effects; and *optional*, in which the desirable effects of pharmacogenetic-based dosing are closely balanced with undesirable effects and there is room for differences in opinion as to the need for the recommended course of action. Each recommendation also includes an assessment of its usefulness in pediatric patients. 

CPIC guidelines currently focus on gene-drug pairs for which at least one of the prescribing recommendations is actionable (*e.g*., recommendation to alter a dose or consider an alternative drug based on the genotype- phenotype relationship). For these and many other gene-drug pairs, PharmGKB also contains clinical annotations that are genotype-based summaries of the association between a drug and a particular variant. Each clinical annotation is assigned a level of evidence depending on population, replication, effect size and statistical significance (reviewed in [17]).

CPIC is considering developing gene-drug pair “evaluations,” as opposed to guidelines, for cases in which the evidence is weak, the prescribing recommendations are optional, or the weight of the evidence does not support altering prescribing based on genetics. As more and more preemptive genotyping is performed, providing guidance to clinicians linking gene variation to drug response and how or whether to use genetic test results, even when recommendations are not strong, becomes more relevant to practice.

### Development of Guideline Text

All CPIC guidelines adhere to a standard format and contain the necessary information a clinician would need to translate patient-specific diplotypes for each gene into clinical phenotypes (*e.g*., CYP2D6 poor metabolizer) or drug prescribing groups (*e.g*., carrier of HLA-B*15:02). The therapeutic recommendations are based on these predicted phenotypes or groupings. As such, assignment of phenotype based on genotype is an important step in the CPIC guideline development process, especially because phenotype assignment is variable in the literature. Each guideline contains a table that assigns likely function to relevant alleles and phenotypes based on possible genotypes (see [1] for example). Also, the online supplementary material may contain additional tables describing the association between specific allelic diplotypes and phenotypes. 

The guideline text also includes background information regarding the gene, drug, and therapeutic recommendation. The gene-based information contains a brief description of the gene, information regarding genetic test interpretation and available genetic testing options, including incidental findings based on potential pleiotropic effects such as “X diseases or conditions that have/have not been linked to variation in gene Y, unrelated to medication use,” and an “other considerations” section for critical issues about the gene that do not easily fit into other sections. The drug-based information contains a brief description of the drug, background linking genetic variability to variability in drug-related phenotypes, and rationale and evidence supporting the therapeutic recommendation. The section “other considerations” includes caveats that non-genetic considerations are also important for prescribing decisions. Examples include drug-drug interactions, epistasis, route of administration, complementary clinical laboratory tests such as the determination of an enzyme activity or metabolite levels, as well as variation in additional genes that may contribute to drug response and adverse drug reactions. 

Each guideline also contains a section on potential benefits and harm for the patient. This section discusses the consequences, toxicities or adverse drug reactions that may be avoided by pharmacogenetic-based dosing as well as any potential risks from incidental findings or use of alternative drugs or dosing strategies (*e.g.,* differences in efficacy). Another potential risk discussed in CPIC guidelines is a possible error in genotyping, as these errors could stay in the medical record for the life of the patient.

Each guideline also contains a “caveats” section, discussing the appropriate use and/or potential misinterpretation of genetic tests. For example, conventional pharmacogenomic genotyping classifies alleles as functional by exclusion, that is, when no common inactivating variants are identified. However, a plethora of rare loss-of-function and reduced-function alleles exist, and unless the relevant pharmacogenetic genes are adequately sequenced, there is a chance that a patient assigned a “wild-type” allele may in fact harbor a rare loss-of-function variant.

Any further clinical information or explanations the writing committee agrees may be pertinent to include may be added to the supplemental information. However, economic analyses and recommendations or guidance for use of other laboratory tests are generally beyond the scope of these guidelines, as CPIC guidelines are written to focus on how to use genotyping data once available. Furthermore, CPIC guidelines also include a disclaimer that the guidelines are limited in scope, providing prescribing recommendations based only on genotype/phenotype, and are not considered inclusive for all clinical considerations affecting prescribing. Disclaimers like this are common across all clinical practice guidelines. 

### Internal and External Review, Comment, and Approval Process

Once the writing committee has completed and approved a draft guideline, the draft guideline is circulated to the CPIC co-leaders and coordinator for content review. The guideline is reviewed for compliance with the CPIC Standard Operating Procedures and required format. The guideline draft is then discussed on a CPIC conference call with all CPIC members and circulated to the members for further review and approval. At each stage, feedback is considered for incorporation into the guideline and/or revision of the guideline, as supported by the available evidence and expert clinical judgment of the senior author and writing committee. Finally, the guideline manuscript under goes typical external scientific peer-review by the journal prior to publication. Current agreements with the American Society for Clinical Pharmacology and Therapeutics give the journal *Clinical Pharmacology and Therapeutics* the first right of refusal for publication of CPIC guidelines; as part of this agreement, the guidelines are freely posted to PharmGKB immediately upon publication. In general *Clinical Pharmacology and Therapeutics *uses a minimum of two external expert peer-reviewers and an editorial board member with content expertise as reviewers for each CPIC guideline.

### Periodic Review and Guideline Updates

On an ongoing basis, CPIC members or others may respond to important new information that would modify prescribing recommendations by updates posted on PharmGKB. Importantly, CPIC guidelines are also systematically reviewed for updates on a regular basis, currently scheduled every two years. The writing process for each guideline update is similar to the process used for the original guideline. However, the degree of guideline modification may vary, ranging from a statement indicating that the original guideline and recommendations are maintained to minor or major revision of the guideline. A literature review of new publications and any other relevant new information is incorporated into all guideline updates. Updated guidelines and supplements are posted on PharmGKB.

### Guideline Dissemination

Once published online, each CPIC guideline is also incorporated online at PharmGKB (www.pharmgkb.org) and announced to the pharmacogenetic community via a blog posting. Each gene-drug pair has its own page with interactive therapeutic recommendations, enabling the user to select a particular genotype or diplotype to view the specific therapeutic recommendation. A summary table is included that provides phenotype assignments based on genotypes, implications for use of the drug based on phenotype/genotype, and the gene-drug based recommendations. The full published article and supplements are also available for download on PharmGKB. CPIC pharmacogenetic guidelines are fully integrated into the PharmGKB database and are available as a JSON (Java Script Object Notation) download for integration with other systems, as well as linking to gene and drug pages with further relevant pharmacogenetic information such as FDA drug labels. CPIC guidelines are listed on PharmGKB along with guidelines from other professional societies, enabling comparison with other known existing guidelines. 

## COMPARISON OF CPIC GUIDELINES TO THE INSTITUTE OF MEDICINE’S STANDARDS FOR DEVELOPING TRUSTWORTHY CLINICAL PRACTICE GUIDELINES

The IOM has a long standing interest in CPG development that extends back to the 1990s when two IOM reports were published on the topic [19, 20]. Subsequently in 2011, the IOM published recommendations resulting from a consensus study ordered by the United States Congress (and subsequently approved by the governing board of the National Research Council) and funded by the Agency for Healthcare Research and Quality (AHRQ). Intended to promulgate a set of standards for the development of rigorous, trustworthy guidelines, this report details several critical factors deemed essential to sound practice guidelines, including transparency; conflict of interest; guideline development group composition; the intersection between CPGs and systematic reviews (SRs); foundations for and strength of recommendations; external review; and process for updating. The over-arching intent of the report was to reinforce critical work by numerous independent researchers as well as consumers (users) of CPGs by connecting expert clinical consensus to evidence-based guideline development. Overall, the CPIC guideline development process closely mirrors the eight IOM standards. 

Table **2** compares the CPIC guideline development process to the IOM standards. The CPIC process aligns well, with the exception of standard 4, which highlights the opportunity created at the intersection between CPGs and SRs. According to the IOM report, the gold standard method consists of ‘complete interaction’ between CPG groups and SR teams, with a superior product resulting from a process involving the same team of individuals to conduct the SR, grade the evidence, and subsequently generate the guideline(s). Authors of CPIC guidelines have often been involved in SRs for their gene/drug topic, but there is no concerted effort to involve the same team of authors. The updated American College of Chest Physicians (ACCP) Antithrombotic Therapy Guideline (2012) provides an example for operationalizing this standard [21]. The ACCP guideline development committee decided *a priori* to use research methodologists to conduct all evidence review and grading rather than clinicians or experts in antithrombotic therapy research. Of note, the research methodologists involved were aided by existence of many published clinical trials (for many of the recommendations) used similar methods and outcome measures. Evidence review was systematically conducted using an established framework, and studies were assessed and summarized using criteria from the Cochrane Collaboration. When evidence grading was completed, the writing group voted to approve or not approve recommendation statements drafted from the graded evidence. In comparison, because of the nature of pharmacogenomic studies, the CPIC guideline development process often relies on published results of pharmacogenomics studies, observational cohort studies providing post-hoc analysis of clinical trials, and preclinical experimental work, some of which can vary with respect to methodological rigor and study outcomes [18]. This variability hampers the development of SRs and subsequent meta-analyses in pharmacogenomics. If comprehensive SRs are undertaken in the future, they can be incorporated into future CPIC guideline updates. 

Other differences between the CPIC guideline process and IOM standards are worth noting. CPIC requires full disclosure of all workgroup members’ potential conflict of interest. While current policies do not explicitly require those with conflict of interest to divest interests or ensure that those with conflict of interest are in the minority of the workgroup, a review of the extant guidelines shows that in most cases the majority of guideline authors declare no conflict of interest. All proposed guidelines are reviewed by CPIC members and an independent peer review managed by journal editors, which act to ameliorate concerns about undue influence of conflict of interest. Another unique aspect of the CPIC guidelines that may mitigate concerns regarding conflict of interest is that CPIC guidelines focus on how to use pharmacogenomic information available to the clinician, not whether to order a genetic test. In this way the guidelines do not recommend a test *per se* which further limits the potential impact of conflict of interest. 

CPIC guidelines are all posted in draft form on CPIC’s website for comment by CPIC members prior to submission of the guideline for publication. At present, CPIC does not actively include a mechanism for comment by the general public either through representation on the guideline workgroup or through a public comment period prior to publication, a feature that is recommended by the IOM. The selection and prioritization of guideline topics incorporates the patient perspective indirectly through the surrogate of the FDA black box warning, but this does not meet the letter of the IOM recommendations. Personal communication and experience from several authors involved in other professional society guideline efforts indicate that CPIC is not alone in evaluating how to meet this IOM standard. CPIC will continue to discuss and evaluate the best approach to expand opportunities for public comment.

## CONNECTING THE GUIDELINES TO PRACTICE

An ultimate goal for CPIC guidelines is to guide clinicians to make patient care decisions for specific drugs when genetic results are available. Electronic health records (EHRs) and clinical decision support (CDS) are essential to implementation of pharmacogenomics [14, 15, 22], and CPIC is working to position the guidelines to effectively guide gene based prescribing in an electronic environment, especially the recommendation of each guideline that details the genetic result and the appropriate clinical action. Efforts are underway to make CPIC guidelines more machine-readable, including making the guidelines available in various file formats (*e.g*., JSON format).

To address the growing interest in the informatics aspects of CPIC guidelines and clinical implementation of pharmacogenetics, CPIC created an informatics working group in 2013. The working group’s goal is to support the adoption of the CPIC guidelines by identifying, and resolving where possible, potential technical barriers to the implementation of the guidelines within a clinical electronic environment. Because detailed information that translates each result from genotype to phenotype to clinical recommendation is needed to implement pharmacogenetics into patient care, the informatics working group’s initial focus is creating translation tables, which complement similar data gathered by the Translational Pharmacogenetics Project (TPP), a network wide PGRN venture [23]. Initial versions of these tables have been posted on PharmGKB for *CYP2D6, CYP2C19, *and *TPMT *(http://www.pharmgkb.org/page/tppTables)*, *and processes to develop and maintain each table as part of the CPIC guideline update process are being developed. Other informatics priorities for CPIC include encouraging vendors that produce clinical electronic references and EHRs to include CPIC guidelines and developing recommendations for CDS based on the guidelines. Long term opportunities include working with standards development organizations to create content that supports the CPIC guidelines and pharmacogenetics, such as terms that properly define metabolizer status and other pharmacogenetic phenotypes, and generating computable representations of the CPIC guidelines using formal knowledge representation and standard terminologies. Ultimately, these efforts will position the CPIC guidelines to work effectively in our increasingly electronic health care system. 

A number of methods are used to distribute and support uptake of CPIC guidelines after publication. As discussed above, all guidelines are posted on the PharmGKB website, linked to pages for the gene and the drug that also provide other relevant information. Furthermore, all guidelines are cited in PubMed and classified as a “practice guideline,” a method used to organize and index published clinical practice guidelines in PubMed. The final published full text guidelines are also freely available in the NIH’s PubMed Central^®^ repository. Moreover, the NIH’s Genetic Testing Registry, which provides a central location for voluntary submission of genetic test information by providers, provides links to CPIC guidelines based on condition/phenotype (*e.g*., thiopurine methyltransferase deficiency; http://www.ncbi.nlm.nih.gov/gtr/conditions/C0342801/). Recently, CPIC guidelines have been accepted for inclusion to the AHRQ-sponsored National Guideline Clearinghouse (www.guidelines.gov) and summaries of the guidelines, including the prescribing recommendation, are freely available to the public from this site as well. 

Indicators for the growing uptake of CPIC guidelines are emerging. The first eight CPIC guidelines and the first guideline update [2-9, 11] have already been cited numerous times in other published literature. The American Society of Health-System Pharmacists (ASHP), a national professional organization for pharmacists with over 40,000 members, has formally endorsed the *CYP2C19*/clopidogrel and *CYP2D6*/codeine CPIC guidelines (http://www.ashp.org/menu/PracticePolicy/PolicyPositionsGuidelinesBestPractices/BrowsebyDocumentType/EndorsedDocuments.aspx). Other professional organizations have also indicated an interest in formally endorsing CPIC guidelines.

## CONCLUSIONS

Adoption of pharmacogenetic tests in routine clinical practice has been sparse. One major barrier to clinical implementation is the lack of clear, curated, peer-reviewed pharmacogenetic guidelines. CPIC guidelines represent a critical step enabling the translation of clinical genetic test results into actionable prescribing decisions. All CPIC guidelines adhere to a standard format and contain the necessary information to help clinicians translate patient-specific diplotypes for each gene into clinical phenotypes or drug prescribing groups. Our experiences indicate that key factors for successful development and adoption of these guidelines include: (1) adhering to standard systems for grading levels of evidence and for assigning strength to each prescribing recommendation;(2) close collaboration among clinicians, scientists, pharmacologists, and informatics experts; (3) provision of clear and specific recommendations for prescribing and (4) converting the guidelines into machine readable formats for incorporation into electronic medical records and linking to clinical decision support tools. The underlying principle, that CPIC guidelines provide recommendations on how to use but not whether to order genetic tests, has allowed the guideline development process to focus on practical aspects of implementing pharmacogenomics into clinical care. 

## Figures and Tables

**Table 1.** Standard format of CPIC guidelines.

**Introduction**
**Literature Review Process**
**Gene(s)**
*Background*
*Genetic Test Interpretation*
*Available Genetic Test Options*
*Incidental Findings*
*Other Considerations*
**Drug(s)**
*Background*
*Linking genetic variability to variability in drug-related phenotypes*
*Dosage Recommendations/Therapeutic Recommendations*
*Recommendations for Incidental Findings*
*Other Considerations*
**Potential Benefits and Risks for the Patient**
**Caveats: Appropriate Use and/or Potential Misuse of Genetic Tests**
**Disclaimer**
**Assignment of likely [gene] phenotypes based on genotypes (Table 1^a^ in CPIC guidelines)**
**Recommended Prescribing of [drug/s] by [gene] phenotype (Table 2^a^ in CPIC guidelines)**

a See [2] for examples.

**Table 2.** Comparison of CPIC guidelines to the IOM’s Standards for Developing Trustworthy Clinical Practice Guidelines.

IOM Standard	CPIC Guidelines

**Establishing transparency**	

1.1 The process for developing a clinical practice guideline (CPG) –including funding – should be explicitly described and publicly accessible.	*In the initial article articulating the need for pharmacogenetics guidelines Relling and Klein outlined the CPIC process [1]. This article does not describe funding, but CPIC funding (currently via NIGMS) is fully and publicly disclosed in each guideline and on its webpage on PharmGKB (http://www.pharmgkb.org/page/cpic and http://www.pgrn.org/display/pgrnwebsite/PGRN+Home). The current publication provides additional transparency for the CPIC guideline development process. *

**Management of conflict of interest (COI)**	

2.1 Prior to selection of the Guideline Development Group (GDG), individuals being considered for membership should declare all interests and activities potentially resulting in COI with development group activity, by written disclosure to those convening the GDG.	*The CPIC authorship plan states that all authors should declare all interests and activities potentially resulting in COI by written disclosure to the CPIC Steering Committee and writing committee before the approval of the authorship plan. This includes all possible conflicts, including NIH funding that could be interpreted to indicate that authors are “advocates” of the enclosed recommendations, as well as any sources of revenue from patents or stock ownership. All spouses/family members are included in declarations and all COI will be reported in the guideline manuscript.*

2.2 All COI of each GDG member should be reported and discussed by the prospective development group prior to the onset of their work.	
Each panel member should explain how their COI could influence the CPG development process or specific recommendations.	

2.3 Members of the GDG should divest themselves of financial investments they or their family members have in, and not participate in marketing activities or advisory boards of, entities whose interests could be affected by CPG recommendations.	*CPIC does not require divestiture. All authors are asked to include all possible conflicts, including NIH funding, which could be interpreted to indicate that authors are “advocates” of the enclosed recommendations, as well as any sources of revenue from patents, stock ownership, etc and inclusion of spouses/family members in declarations.*

2.4 Whenever possible GDG members should not have COI. In some circumstances, a GDG may not be able to perform its work without members who have COIs, such as relevant clinical specialists who receive a substantial portion of their incomes from services pertinent to the CPG. Members with COIs should represent not more than a minority of the GDG.	*All authors declare all interests and activities potentially resulting in COI by written disclosure to the CPIC Steering Committee and writing committee before the approval of the authorship plan. Included are all possible COI including spouses/family members in declarations, NIH funding that could be interpreted to indicate that authors are “advocates” of the recommendations, as well as any sources of revenue from patents, stock ownership, etc. All COI are reported in the guideline manuscript. Though it is not feasible to exclude authors with any COI, the CPIC Steering Committee has successfully managed COIs for the GDGs and overall, authors with COIs have constituted a minority of authors of CPIC guidelines. *
The chair or co-chairs should not be a person(s) with COI. Funders should have no role in CPG development.	

**Guideline development composition**	

3.1 The GDG should be multidisciplinary and balanced, comprising a variety of methodological experts and clinicians, and populations expected to be affected by the CPG.	*All guidelines include clinicians and scientists with varying areas of expertise. The CPIC coordinator and/or PharmGKB Scientific Curator serve as the methodological expert on each guideline writing committee. This is highlighted in the authorship plan.*

3.2 Patient and public involvement should be facilitated by including (at least at the time of clinical question formulation and draft CPG review) a current or former patient and a patient advocate or patient/consumer organization representative in the GDG.	*CPIC does not formally consult patients or consumers; however, all draft guidelines are distributed to all members of CPIC.*

3.3 Strategies to increase effective participation of patient and consumer representatives, including training in appraisal of evidence, should be adopted by GDGs.	*This remains an area of opportunity. Future CPIC guidelines may include a mechanism for engaging patient and consumer stakeholders.*

**Clinical practice guideline – systematic review intersection**	

4.1 CPG developers should use systematic reviews that meet standards set by the Institute of Medicine’s Committee on Standards for Systematic Reviews of Comparative Effectiveness Research.	*CPIC meets some but not all of these standards. Experts on CPIC authorship teams have often been involved in writing related systematic reviews of the drug(s) or gene(s) of interest. Due to methodological limitations inherent to many studies evaluating pharmacogenomic applications to clinical therapeutics, there often are not pre-existing systematic reviews of comparative effectiveness.*

4.2 When systematic reviews are conducted specifically to inform particular guidelines, the GDG and systematic review team should interact regarding the scope, approach, and output of both processes.	

**Establishing evidence foundations for and rating strength of recommendations**	

For each recommendation, the following should be provided:	*All CPIC guidelines adhere to this standard.*
An explanation of the reasoning underlying the recommendation, including: A clear description of potential benefits and harms. A summary of relevant available evidence (and evidentiary gaps), description of the quality (including applicability), quantity (including completeness), and consistency of the aggregate available evidence. An explanation of the part played by values, opinion, theory, and clinical experience in deriving the recommendation. A rating of the level of confidence in (certainty regarding) the evidence underpinning the recommendation. A rating of the strength of the recommendation in light of the preceding bullets. A description and explanation of any differences of opinion regarding the recommendation.	

**Articulation of recommendation**	

6.1 Recommendations should be articulated in a standardized form detailing precisely what the recommended action is and under what circumstances it should be performed.	*In published CPIC guidelines, Table 2 articulates recommendations in a standard format.*

6.2 Strong recommendations should be worded so that compliance with the recommendation(s) can be evaluated.	*In published CPIC guidelines, Table 2 articulates recommendations in a standard format such that compliance with the recommendation could be evaluated.*

**External review**	

7.1 External reviewers should comprise a full spectrum of relevant stakeholders, including scientific and clinical experts, organizations (e.g., health care, specialty societies), agencies (e.g., federal government), patients, and representatives of the public.	*All CPIC guidelines in draft form are open to review by all CPIC members; however, there is no current process to facilitate review by patient stakeholders. *

7.2 The authorship of external reviews submitted by individuals and/or organizations should be kept confidential unless that protection has been waived by the reviewer(s).	*External reviews of CPIC drafts are coordinated by the journal editorial staff during the publication process and not facilitated by CPIC.*

7.3 The GDG should consider all external reviewer comments and keep a written record of the rationale for modifying or not modifying a CPG in response to reviewers’ comments.	*Drafts of CPIC guidelines containing CPIC member feedback are stored in order to track modifications and the rationale for responses to specific comments.*

7.4 A draft of the CPG at the external review stage or immediately following it (i.e., prior to the final draft) should be made available to the general public for comment. Reasonable notice of impending publication should be provided to interested public stakeholders.	*This remains an area of opportunity. Future CPIC guidelines may include a mechanism for engaging public stakeholders.*

**Updating**	

8.1 The CPG publication date, date of pertinent systematic evidence review, and proposed date for future CPG review should be documented in the CPG.	*All CPIC guidelines are evaluated for updates approximately every two years and this is stated in the CPIC position paper and each guideline. In addition, PharmGKB updates and curates information on an ongoing basis and has the ability to update CPIC guidelines posted on its site and post alerts regarding any changes.*

8.2 Literature should be monitored regularly following CPG publication to identify the emergence of new, potentially relevant evidence and to evaluate the continued validity of the CPG.	

8.3 CPGs should be updated when new evidence suggests the need for modification of clinically important recommendations. For example, a CPG should be updated if new evidence shows that a recommended intervention causes previously unknown substantial harm, that a new intervention is significantly superior to a previously recommended intervention from an efficacy or harms perspective, or that a recommendation can be applied to new populations.	
